# Pulsed electromagnetic fields as a promising therapy for glucocorticoid-induced osteoporosis

**DOI:** 10.3389/fbioe.2023.1103515

**Published:** 2023-03-03

**Authors:** Tianxiao Zhang, Zhiliang Zhao, Tiantian Wang

**Affiliations:** ^1^ Innovation Center for Wound Repair, West China Hospital, Sichuan University, Chengdu, China; ^2^ Key Laboratory of Rehabilitation Medicine, West China Hospital, Sichuan University, Chengdu, Sichuan, China; ^3^ Institute of Rehabilitation Medicine, West China Hospital, Sichuan University, Chengdu, Sichuan, China

**Keywords:** GIOP, PEMFs, bone cells, angiogenesis, mechanism

## Abstract

Glucocorticoid-induced osteoporosis (GIOP) is considered the third type of osteoporosis and is accompanied by high morbidity and mortality. Long-term usage of glucocorticoids (GCs) causes worsened bone quality and low bone mass *via* their effects on bone cells. Currently, there are various clinical pharmacological treatments to regulate bone mass and skeletal health. Pulsed electromagnetic fields (PEMFs) are applied to treat patients suffering from delayed fracture healing and non-unions. PEMFs may be considered a potential and side-effect-free therapy for GIOP. PEMFs inhibit osteoclastogenesis, stimulate osteoblastogenesis, and affect the activity of bone marrow mesenchymal stem cells (BMSCs), osteocytes and blood vessels, ultimately leading to the retention of bone mass and strength. However, the underlying signaling pathways *via* which PEMFs influence GIOP remain unclear. This review attempts to summarize the underlying cellular mechanisms of GIOP. Furthermore, recent advances showing that PEMFs affect bone cells are discussed. Finally, we discuss the possibility of using PEMFs as therapy for GIOP.

## 1 Introduction

Glucocorticoids (GCs) are used as a treatment to suppress inflammation, as well as for various inflammation-mediated diseases, including ankylosing spondylitis (AS) and rheumatoid arthritis (RA) (Crawford et al., 2003). However, prolonged GC therapy can cause glucocorticoid-induced osteoporosis (GIOP), which is considered the third type of osteoporosis (Schorlemmer et al., 2005). At present, the main negative effects of excess GCs on the skeleton are considered to be exerted on bone cells directly, affecting osteoblasts, osteoclasts, osteocytes, and bone marrow mesenchymal stem cells (BMSCs) (Wang et al., 2018). BMSCs have the potential to differentiate into different kinds of cells (Chamberlain et al., 2007). In bone tissue, there is a dynamic balance between differentiation into adipocytes and osteoblasts. The balance plays an important role in lipid metabolism and bone homeostasis. In addition, some groups reported that excessive GC use could disturb the balance between lipid metabolism and bone remodeling (Takano-Murakami et al., 2009) by upregulating adipogenesis and downregulating osteogenesis of BMSCs (Yin et al., 2006). GCs increase the expression of adipogenesis-associated genes, such as peroxisome proliferator‐activated receptor‐γ2 (PPAR‐γ2), but decrease osteogenic gene expression, especially Runt‐related transcription factor 2 (Runx2) (Li et al., 2005). Moreover, chronic GC treatment may lead to metabolic defects, resulting in lower serum insulin levels, higher blood glucose, and enhancement of visceral obesity (Lee et al., 2007). These factors might be related to GC-induced obesity and diabetes, which in turn cause severe osteoporosis.

At present, for the treatment of GIOP, calcium and vitamin D are the basic treatments, while bisphosphonates and terlipatide are the main treatment drugs, which increase bone density and reduce fracture risk in GIOP patients. Calcitonin is mainly used to relieve bone pain and is applicable to patients who do not tolerate or have contraindications to the above drugs (Pereira et al., 2020). However, long-term use of these antiosteoporosis drugs can also cause potential side effects, including osteonecrosis of the jaw, gastrointestinal complaints, and typical subtrochanteric or diaphyseal femoral fractures (Canalis et al., 2007). In addition to pharmacotherapy, physical therapy is a non-invasive and safe biophysical countermeasure, which should be the highest recommendation in clinical practice. Pulsed electromagnetic fields (PEMFs) have been proven to exert anti-inflammatory effects and are efficient in treating many bone disorders, including fresh fractures, non-union and delayed fractures, osteoporosis, diabetic osteopenia, and osteonecrosis (Liu et al., 2013; Liu et al., 2017; Wang et al., 2019a; Wang et al., 2019b). At present, the application of PEMFs to GIOP is not yet popularized in clinical practice, but some animal experiments have shown promising results. For example, one group found that PEMF therapy might alleviate bone loss and reduce serum lipid levels without negative effects in GIOP rats. The process depends on the Wnt/β-catenin signaling pathway (Jiang et al., 2016). Our group reported that PEMFs eliminated senescent cells to rescue bone loss in GIOP mice (Wang et al., 2021; Wang et al., 2022a). Furthermore, PEMFs also eliminate the side effects of GCs on osteoblasts (Esmail et al., 2012). We can infer that PEMF treatment may be an effective, safe, and non-invasive therapy for GIOP and might provide some potential benefits for patients with GIOP.

In this review, we first summarize the underlying cellular mechanisms of GIOP. Moreover, recent advances have shown that PEMFs affect bone cells. Finally, we discuss the possibility of using PEMFs as a therapy for GIOP.

## 2 PEMFs

PEMFs are low-frequency magnetic fields with a specific amplitude and waveform characterized by a stable variation in the amplitude of the magnetic field over time. In exposed tissue, PEMFs create a secondary electric field, which is similar to the one naturally generated during the conversion of mechanical energy into electrical energy (Zhu et al., 2017). Two methods, inductive or capacitive coupling, can be used to apply PEMFs in biological tissues. In direct capacitive coupling, the electrodes must be placed on the tissue, but in inductive coupling (non-direct capacitive coupling) they may not be in direct contact with the tissue. The reason is that the electric field produces a magnetic field, and then a current can be produced in the conductive tissues in the body (Ross et al., 2019). PEMFs are a non-invasive method of physical therapy for skeletal diseases. In 1978, Martin found that PEMFs have therapeutic effects in osteoporosis (Matsunaga et al., 1996). Recently, PEMFs have also been proven to improve bone mineral density in the spine, distal radius, and knee in osteoporosis patients (Roozbeh and Abdi, 2018). PEMFs have widespread application with rapid effects, easy operation, and no adverse effects. It has been demonstrated that PEMF therapy is a safe, non-invasive, and easy method to treat inflammation, dysfunctions, and pain related to osteoarthritis (OA) and RA (Ganesan et al., 2009). Waldorff et al. (2017) also reported that PEMF increased the speed of bone healing when used as a treatment for fracture patients. Additionally, PEMFs have been proven to be beneficial in enhancing bone mechanical strength and improving bone microstructure by promoting bone formation and suppressing bone resorption in a study of a New Zealand white rabbit model of osteoporosis (Qian et al., 2021). Moreover, PEMF therapy attenuated bone resorption, enhanced BMD, and promoted osteogenesis in rats with disuse osteoporosis. In an ovariectomy (OVX)-induced osteoporosis mouse model, a PEMF modulated the anabolic and catabolic activity of bone, upregulated the expression of osteogenesis-related genes, and promoted trabecular bone formation (Wang et al., 2022b).

## 3 Bone

Bone is a metabolically-active tissue related to the physiological processes of locomotion, providing structural support and movement facilitation by providing storage of minerals and growth factors, regulation of mineral and acid–base homeostasis, protection of important structures, muscle levers, and a site for hematopoiesis. A central marrow space surrounded by periosteum and bone tissue is the general structure of a long bone (Buck and Dumanian, 2012). Bone remodeling is a process that involves replacing old bone with newly-formed bone periodically at the same location and is involved in osteoporosis (Siddiqui and Partridge, 2016). Bone is composed of various cell types that undergo continuous remodeling (Raggatt and Partridge, 2010; Buck and Dumanian, 2012; Siddiqui and Partridge, 2016).

BMSCs, or marrow stromal cells (MSCs), were confirmed to be precursors for several different cell lineages, such as chondrocytes, osteoblasts, adipocytes, myoblasts, and fibroblasts (Kfoury and Scadden, 2015), which are regulated by Wnt signaling pathways and bone morphogenetic proteins (BMPs) (Chen et al., 2016). Under the stimulation of multiple factors, activated osteoblasts proliferate in large numbers at the depression of bone resorption, secrete a variety of bone formation-related proteins, combine with extracellular crystalline hydroxyapatite and other inorganic components to form mature bone matrix, and gradually mineralize to form new bone (Kylmaoja et al., 2016). Osteoclasts are the primary functional cells of bone resorption and play a vital role in bone growth, development, repair and reconstruction. Osteoclasts, which express receptor activator of nuclear factor kappa-B (NF-κB) ligand (RANKL) and macrophage colony stimulating factor (M-CSF), originate from the blood mononuclear macrophage system and are special terminally-differentiated cells. Fusion of mononuclear precursor cells forms giant multinucleated cells in various ways (Xu and Teitelbaum, 2013). Mature osteoclasts are multinuclear cells produced by the fusion of tartrate-resistant acid phosphatase-positive (TRAP^+^) mononuclear cells, termed preosteoclasts (POCs), and are the main source of platelet-derived growth factor-BB (PDGF-BB) (Boyle et al., 2003; Xu and Teitelbaum, 2013; Kusumbe et al., 2014; Xie et al., 2014; Zhen et al., 2021). Type H vessels are located abundantly in the metaphysis adjacent to the growth plate, are linked with bone formation, and are double-positive for CD31 and Emcn (Peng et al., 2020a; Zhen et al., 2021). PDGF-BB, released by POCs, promotes angiogenesis of type H vessels and osteogenesis (Peng et al., 2020a). Osteocytes are terminally-differentiated osteoblasts embedded in the bone matrix, which are a major source of sclerostin (SOST) and RANKL, regulating osteoblast and osteoclast formation, respectively (Goldring, 2015; Wang et al., 2019c). Additionally, osteoclasts lay down minerals and create the collagen-rich bone matrix, transforming mechanical inputs into biochemical signals (Bidwell et al., 2008).

GIOP can be induced as a primary side effect of the application of GCs based on various mechanisms. It is reported that GCs might be toxic to genes related to cell regulation (osteoblasts, etc.) through combining with the promoter region of GC response elements, eventually resulting in changes of protein regulation and synthesis (Adami and Saag, 2019). Moreover, GCs can be harmful to bone formation *via* two main pathways: enhancing expression of peroxisome proliferator-activated receptor gamma 2 (PPARγ2) and suppressing the typical Wnt/β catenin signaling pathway (Adami and Saag, 2019). Previous evidence has proven that GCs induce apoptosis of osteocytes and osteoblasts, impairing the function and differentiation of osteoblasts directly. In addition, another study showed that T cells could also lead to bone loss through the RANKL pathway and regulation of CXCL10, resulting in GIOP (Song et al., 2020). The details of GC targeting of bone are discussed below.

## 4 Potential targets of PEMFs in GIOP

### 4.1 BMSCs

A faulty early mesenchymal precursor, presumably an MSC, will cause a reduction in the production of osteoprogenitor cells and may be responsible for a variety of musculoskeletal problems, including osteoporotic syndromes (Bonyadi et al., 2003). BMSCs are a critical cellular target of GCs in developing bone turnover (Shen et al., 2018). GC treatment increases the number and size of bone marrow adipocytes, switching the fate of BMSCs from osteogenesis to adipogenesis (Bujalska et al., 1999). This process has been demonstrated to depend on transactivation of CCAAT/enhancer binding protein in murine stromal cells (Pereira et al., 2002; Canalis et al., 2004) accompanied by an upregulation of PPARγ2 and downregulation of Runx2 (Canalis et al., 2007), leading to increased bone marrow adipose tissue, fewer mature osteoblasts and decreased cancellous bone (Weinstein and Manolagas, 2000). In addition, GCs stimulate preadipocyte conversion to mature adipocytes, resulting in the hyperplasia of adipose tissue. In a GC-treated model, a two-fold increase was found in the cancellous adipocyte area (Weinstein and Manolagas, 2000). Stimulation of osteogenic MSCs is a relatively new concept in medicine that could potentially be achieved by the use of PEMFs. PEMFs have the potential to prevent aberrant and promote healthy MSC function. PEMFs (75 Hz, 1.5 mT, 28 days) have been shown to exert suppressive effects on the expression of adipogenic genes (Jansen et al., 2010; Lu et al., 2015) and induce osteogenesis through the enhancement of ALP activity and the expression of Runx2 in BMSCs (Ongaro et al., 2014), accompanied by a delayed increase in cell proliferation. Stimulation of osteogenesis through application of a PEMF alleviated bone loss in GIOP models (Wang et al., 2022a). The Wnt/β-catenin pathway might be involved in this process. For example, GCs disturb the BMSC differentiation balance by upregulating adipogenesis-related genes and downregulating osteogenesis-associated genes by suppressing the Wnt/β-catenin pathway (Li et al., 2013). The mRNA and protein expression levels of Wnt10b, LRP5, and β-catenin were significantly upregulated in GIOP rats after PEMF stimulation for 12 weeks (50 Hz, 4.0 mT, 40 min per day), suggesting that the canonical Wnt signaling pathway was activated during PEMF stimulation, which is in agreement with previous reports (Ding et al., 2011; Jing et al., 2013; Jiang et al., 2016). In addition, the mTOR signaling pathway plays a crucial role in a variety of diseases, including GIOP (Wang et al., 2020; Ge and Zhou, 2021). Suppressing mTOR signaling induces osteoblastic differentiation and reduces adipogenic potential (Martin et al., 2015). One group reported that exposure to PEMFs reversed the reduced mineralization of the extracellular matrix (ECM) induced by rapamycin, an inhibitor of TORC1 (receptor of mTOR) (Sarbassov et al., 2006), suggesting that PEMFs might stimulate BMSC commitment to the osteoblast lineage *via* the mTOR pathway (Ferroni et al., 2018). Whether mTOR participates in the rescue of GIOP by PEMFs requires further study.

Recently, cellular senescence, characterized by loss of replicative potential, has been shown to have a crucial role in GIOP (Liu et al., 2021; Wang et al., 2021; Wang et al., 2022c). For example, in young mice, Nestin-expressing (Nestin^+^ cells), a type of MSC in postnatal bones, are primarily of endothelial and osteoblast lineages (Ono et al., 2014) and undergo senescence in response to GCs (Li et al., 2017; Su et al., 2020). In addition, LepR^+^ MSCs of adult mice are also susceptible to GC treatment (Wang et al., 2021). Our group reported that LepR^+^ cells exhibit a senescent phenotype based on flow cytometry and immunostaining analysis (Wang et al., 2021; Wang et al., 2022a). Clearance of senescent cells by PEMF treatment (8 Hz, 3.8 mT, 1 h per day) for 4 weeks rescued GC-induced bone loss (Wang et al., 2021; Wang et al., 2022a). In particular, PEMFs exerted anti-senescence effects on LepR^+^ MSCs through the EZH2–H3K27me3 axis (Wang et al., 2022a).

In conclusion, PEMFs play an important role in regulating the balance of BMSC production and differentiation through various pathways either directly or indirectly. Thus, there is potential to apply PEMFs to the treatment of GIOP in future.

### 4.2 Osteoblast function

The effects of PEMFs on osteoblast function remain debatable; it is well known that PEMFs have a window effect and produce repeatable osteogenic effects (Matsunaga et al., 1996). Different PEMF intensities and different time-points chosen for analysis can cause different effects. However, most studies assumed that PEMFs could enhance osteoblast activity, leading to an increase in cellular differentiation (Diniz et al., 2002).

There are many assumptions related to the mechanism of how PEMFs affect osteoblast lineages in response to GCs. First, osteoblast orientation and morphology can be regulated by PEMFs. A PEMF (60 Hz, 0.7 mT, 24 h) was shown to mediate osteoblast differentiation by inducing morphological changes, making osteoblastic cells smaller, shorter and rounder in comparison to sham treatment (Lee and McLeod, 2000), which should be tested in GIOP. In support of this, our experiments showed that long-term GC treatment caused detrimental effects on osteoblasts, which could be reversed by PEMF therapy (8 Hz, 3.8 mT, 1 h per day) (Wang et al., 2021; Wang et al., 2022a). The Wnt signaling pathway might also account for this. For instance, GCs suppresses the synthesis and release of transcription factors of the Wnt signaling pathway in mature osteoblasts (Mak et al., 2009), such as β-catenin and Runx2, impairing osteoblast differentiation. Specifically, therapeutic concentrations of GCs upregulate the expression of glycogen synthase kinase 3β (GSK-3β), resulting in β-catenin degradation (Wang et al., 2009). Meanwhile, high levels of GCs also promote the expression of Wnt inhibitors such as SOST and DKK1 (Mak et al., 2009). PEMFs have been demonstrated to increase the expression of genes associated with the Wnt signaling pathway, including Wnt1a, Wnt3a, Lrp5, and Lrp6, both *in vivo* and *in vitro*. In addition, a PEMF (50 Hz, 4.0 mT, 40 min per day) also downregulated DKK1, which antagonized the Wnt signaling pathway (Zhou et al., 2015; Jiang et al., 2016) in a rat model. The role of the canonical Wnt signaling pathway was investigated after PEMF treatment in a GIOP model (50 Hz, 4.0 mT, 40 min per day for 12 weeks) (Jiang et al., 2016). A PEMF reversed the decreased expression of Wnt10b, LRP5, and β-catenin induced by GCs (Jiang et al., 2016).

In addition, BMP-2, a regulator of osteoblast differentiation, is suppressed by high concentrations of GCs (Yao et al., 2008). Moreover, therapeutic levels of GCs enhance the expression of BMP-2 antagonists, such as follistatin and Dan family members (Hayashi et al., 2009). Li et al. (2007) showed that a PEMF (7.5 Hz, 108 μT, 20 min per day for 4 days) upregulated the mRNA production of TGF-β, BMP2, osteocalcin, osteoprotegerin, ALP, Runx2, NF-γB ligand, matrix metalloproteinase-l and -3 (Chen et al., 2010), and bone sialoprotein. These studies suggested that osteogenic differentiation of osteoprogenitor cells could be stimulated by PEMFs directly *via* the BMP2 signaling pathway (Schwartz et al., 2008). To clarify the mechanism of PEMF therapy, further studies should evaluate the role of BMP2 in bone loss induced by GCs.

Furthermore, GCs inhibit the synthesis of type I collagen (COL1A), resulting in decreased bone matrix formation *in vitro* (Canalis, 1983; Harris et al., 2013). In addition, PEMFs may not only upregulate genes involved in bone and matrix component formation but also downregulate various genes related to ECM degradation (Sollazzo et al., 2010). Sollazzo et al. (2010) reported that PEMFs (75 Hz, 2 mT, 18 h) increase the expression of genes related to bone formation, including AKTl and HOXA10; genes associated with transduction activation, such as P2RX7 and CALM 1; genes encoding organic ECM components, such as SPARC and COLlA2; and genes correlated with cytoskeletal components, including VCL and FNI. Smith et al. (2004) found that PEMFs (2 min or 1 h) might suppress the expression of genes for matrix degradation, such as downregulation of phosphatase 4 (DUSP4) and matrix metalloproteinase 11 (MMP-11). Although *in vivo* and *in vitro* studies showed promising effects of PEMFs on matrix mineralization, this speculation has not been tested in GIOP models, which need further study in future.

### 4.3 Osteocytes

Recent experiments have reported that osteocytes act as the main targets for excessive GCs in bone (O’Brien et al., 2004). Osteocytes are thought to control the fate of both osteoclasts and osteoblasts (Weinstein et al., 2002; Weinstein, 2007). GCs induce osteocyte autophagy during the initial period (Xia et al., 2010), while prolonged usage of GCs causes osteocyte death, resulting in a decrease in osteocyte number, accompanied by significantly impaired bone quality. However, autophagy induction in osteocytes cannot rescue the negative effects of GCs on bone metabolism (Piemontese et al., 2015). Exposure to high levels of GCs may induce osteocyte apoptosis, causing them to secrete more DKK1 and SOST, thereby suppressing the combination between Wnt and LPR5/-6 (Hayashi et al., 2009), leading to reduced bone formation. On the other hand, dying osteocytes secrete more TNF-α, IL-6, HMGB1, and RANKL to stimulate osteoclastogenesis. Cai found that the GC-treated group contained a considerably higher proportion of apoptotic osteocytes than the control group based on the results of TUNEL immunofluorescence staining, and the PEMF group rescued this progression. Moreover, PEMF partially mitigated the increase in the gene expression of SOST and DKK1, suggesting that PEMFs (15 Hz, 2.0 mT, 2 h per day for 6 weeks) attenuated the apoptosis of osteocytes stimulated by GCs (Cai et al., 2020).

In addition to signaling pathway connections, osteocytes also regulate osteoblasts and osteoclasts *via* gap junction intercellular communication (GJIC), including Cx43, which is negative for osteoclasts and positive for osteoblasts (Wang et al., 2018). Other small molecules, including prostaglandin E2 (PGE2) and nitric oxide (NO), might also be related to the communication between osteoblasts and osteocytes. PGE2 plays an important role in ECM synthesis and osteoblast differentiation, which is stimulated by TGF-β1. Moreover, NO_2_- inhibits osteoblast activity, stimulates apoptosis, and promote bone resorption (Wang et al., 2018). The gap junctions at the tips of osteocyte cytoplasmic processes respond to alterations of the mechanical environment *via* stimulation including mechanical loading, and deliver signals through the osteocyte network to osteoblasts. In response to fluid flow, functional gap junctions between osteocytes and osteoblasts are created, which then stimulate osteoblastic development. Thus, blocking GJIC suppresses mechanical signal transmission from osteocytes to osteoblasts, leading to impairment of osteoblastic differentiation (Loiselle et al., 2013). GCs impair osteocyte–osteoblast communication by triggering Cx43 degradation, causing severe adverse skeletal effects. GCs inhibit β-catenin stabilization and production of cyclooxygenase-2 (COX-2) and PGE2 (Wang et al., 2018). Loiselle et al. (2013) found that PEMFs (15 Hz, 8 h per day, 4 days) upregulated total TGF-β1 and PGE2 in cells of the murine long bone osteocyte-Y4 cell line (Murine Long bone Osteocyte-Y4; MLO-Y4) over time, which is dependent on the prostaglandin mechanism, including COX-1 (Lohmann et al., 2003). In addition, PEMF stimulation mediates NO_2_- in a time-dependent manner (Lohmann et al., 2003). Based on the evidence detailed above, PEMFs might rescue GIOP by mediating the communication between osteoblasts/osteoclasts and osteocytes. In future, more studies are needed to test this hypothesis in GIOP models.

### 4.4 Osteoclasts

Decreased osteoclast number and viability induced by PEMFs might account for the antagonistic effects against GCs (He et al., 2015; Tschon et al., 2018). Specifically, GC treatment stimulates bone resorption, accompanied by the upregulation of osteoclast number and activity observed in humans and mice (Dovio et al., 2004; Yao et al., 2008). Excess GCs stimulate bone resorption directly by extending the lifespan of mature osteoclasts (Lin et al., 2016). For example, compared with normal rats, rats with GIOP have lower ALP levels and higher TRAP levels in serum (Jiang et al., 2016). Jiang et al. found that PEMF (50 Hz, 4.0 mT, 40 min per day) stimulation significantly decreased serum TRAP levels and increased serum ALP levels (Jiang et al., 2016), suggesting that PEMFs could be an efficient therapy for GIOP. The mechanism needs to be clarified.

Over the past several years the RANK/RANKL/OPG system has been shown to play a vital role in bone remodeling (Borsje et al., 2010). Osteocytes and osteoblasts primarily express RANKL, a cell surface protein that combines with a specific receptor (RANK) located on the osteoclast membrane, contributing to osteoclastogenesis. Osteoblast-derived OPG inhibits osteoclastogenesis by suppressing osteoclast maturation (Lacey et al., 1998). GC treatment stimulates the production of RANKL and decreases the expression of the RANKL decoy receptor osteoprotegerin (OPG) (Sivagurunathan et al., 2005). Disturbing the RANKL/OPG ratio results in increased osteoclast activity and bone resorption. Jiang et al. found that the OPG/RANKL ratio, which is decreased in GIOP, improved after PEMF treatment (Jiang et al., 2016), indicating that the OPG/RANK/RANKL signaling pathway might participate in this process. PEMFs inhibit RANKL expression and enhance OPG expression, leading to upregulation of the OPG/RANKL ratio (Jiang et al., 2016). This process might involve activation of the canonical Wnt signaling pathway after PEMF stimulation in GIOP rats.

Proinflammatory cytokines might accelerate osteoclastogenesis and this could be alleviated by PEMF stimulation, which has been demonstrated in other osteoporosis models. For example, Chang et al. (2004) found that in OVX rats, PEMF treatment (7.5 Hz, 0.8 μT, 9 days) inhibited osteoclastogenesis accompanied by reduced levels of interleukin 1 beta (IL-1β), tumor necrosis factor-alpha (TNF-α), and interleukin 6 (IL-6) in primary bone marrow. To clarify the mechanism of PEMF stimulation, further experiments are in progress to evaluate the role of proinflammatory cytokines in bone metabolism in GIOP models.

### 4.5 Blood vessels

The vasculature plays a critical role in the growing skeleton, and angiogenesis is intimately coupled to osteogenesis. As an essential part of skeletal development, osseointegration, and bone formation, the formation of blood vessels is important in transporting growth factors to achieve cell viability and interaction (Diomede et al., 2020). High doses of GCs are known to inhibit angiogenesis and induce osteoporosis and growth failure (Sivaraj and Adams, 2016). Liu reported that GC treatment induced vascular endothelial cell senescence in young mice, and alleviation of this alteration not only improved GC-impaired bone angiogenesis with coupled osteogenesis but also bone loss. GC treatment inhibits ANG, a ribonuclease secreted by metaphyseal osteoclasts, leading to blood vessel cell senescence and bone loss through binding to PLXNB2 in vascular cells (Liu et al., 2021). Peng demonstrated that chronic GC exposure led to reduced POC numbers and PDGF-BB and thus inhibited type H vessel formation, ultimately resulting in osteoporosis, bone growth retardation, and osteonecrosis (Peng et al., 2020b). In addition, exposure to GCs inhibits the angiogenic molecule VEGF (Pufe, 2003; Athanasopoulos et al., 2007) and stimulates the angiostatic glycopeptide thrombospondin-1 (Rae et al., 2009). We can infer that excess GC causes a decrease in bone water volume and skeletal blood flow (Goans et al., 1995; Drescher et al., 2000) and contributes to a reduced mineral apposition rate (Reeve et al., 1988). Recently, our studies found that the type H vessel number was significantly reduced in the GC group compared to the controls, while PEMFs (8 Hz, 3.8 mT, 1 h per day for 4 weeks) maintained this change, suggesting that PEMFs can show angiogenic–osteogenic effects on bone marrow during GC treatment (Wang et al., 2021; Wang et al., 2022a). Another study performed by Wang demonstrated that PEMFs (15 Hz, 2.4–2.6 mT, 1 h per day for 8 weeks) substantially countered OVX-induced bone loss by inducing coupling promotion of osteogenesis and type H vessels in a mouse model. This beneficial effect might be mediated by HIF-1α signaling in type H vessels (Wang et al., 2022b). These results open up new directions for research into the therapeutic effects of PEMFs on the reversal of osteoporosis by targeting angiogenesis. This speculation needs to be tested in GIOP models in both young and adult mice in the future.

## 5 Evidence for therapeutic effects

PEMFs prevent bone loss because of piezoelectrical effects, modulating calcium deposits in bone and regulating mineral metabolism. For example, our team found that PEMFs reduce bone loss in postmenopausal women and improve their pain and balance function (Liu et al., 2013). Moreover, PEMFs improve bone loss in OVX osteoporosis animal models, enhance the biomechanical properties of bone, and inhibit inflammation [!!! INVALID CITATION !!! (3 and 4)]. In addition to OVX-induced bone loss, other groups found that PEMFs also acted as a therapy for osteoporosis induced by diabetes-mellitus and disuse (Chang and Chang, 2003; Liu et al., 2013). At present, the application of PEMFs to GIOP is not yet popularized in clinical applications, but some animal experiments have shown promising results. Jiang et al. (2016) reported that PEMF therapy antagonized the negative effects of GCs in bone by activating the Wnt/β-catenin signaling pathway. Our group found that PEMFs rescued bone loss in GIOP models by eliminating senescent cells (Wang et al., 2021; Wang et al., 2022a). Furthermore, PEMFs eliminate the side-effects of GCs on osteoblasts (Esmail et al., 2012). Based on the evidence, we conclude that PEMF treatment may be an effective, safe, and non-invasive therapy for GIOP and might provide some potential benefits for patients with GIOP.

There are many potential mechanisms that could account for this. Bone mass maintenance is attributed to a balance between osteoblastic bone formation and osteoclastic bone resorption. Numerous studies have reported that the RANKL–RANK signaling and Wnt signaling pathways are two of the most necessary pathways regulating bone quality and bone metabolism. The Wnt/β-catenin pathway might be associated with the protection of osteogenesis by PEMFs antagonizing GCs. For example, GCs disturb the BMSC differentiation balance by upregulating adipogenesis-related genes and downregulating osteogenesis-associated genes by suppressing the Wnt/β-catenin pathway (Li et al., 2013). The mRNA and protein expression levels associated with the Wnt/β-catenin pathway were significantly upregulated in GIOP rats after PEMF stimulation for 12 weeks, indicating that the canonical Wnt signaling pathway was activated during PEMF stimulation (Ding et al., 2011; Jing et al., 2013; Jiang et al., 2016). Meanwhile, high levels of GCs also promote the expression of Wnt inhibitors such as SOST and DKK1 to suppress osteogenesis (Mak et al., 2009). PEMFs not only increase the expression of genes involved in the Wnt signaling pathway, such as Lrp5, Lrp6, Wnt1a, and Wnt3a but also downregulate SOST and DKK1 (Zhou et al., 2015; Cai et al., 2020). Moreover, dying osteocytes are the main sources of SOST and DKK1, indicating that PEMFs could also alleviate osteocyte apoptosis induced by GCs (Ramli and Chin, 2020). With regard to the ECM, chronic GC treatment suppresses mineralization (Canalis, 1983; Wang et al., 2005), which can be reversed by PEMFs (Sollazzo et al., 2010).

In addition to osteogenesis, GCs increase osteoclast survival indirectly by inhibiting OPG production by osteoblastic cells, thereby upregulating available RANKL and inhibiting osteoclast apoptosis. Jiang et al. (2016) found that the OPG/RANKL ratio, which decreased in the GIOP group, improved after PEMF treatment, indicating that the OPG/RANK/RANKL signaling pathway might participate in this process. PEMFs inhibit RANKL expression and enhance OPG expression, leading to an upregulation in the ratio of OPG/RANKL (Jiang et al., 2016). Another osteoclast population, termed POCs, positively regulate osteogenesis by regulating angiogenesis. Chronic GC treatment has been demonstrated to lead to reduced POC numbers and thus inhibition of type H vessel formation, ultimately resulting in osteoporosis, bone growth retardation, and osteonecrosis (Peng et al., 2020b). In addition, exposure to GCs inhibits the angiogenic molecule VEGF (Pufe, 2003; Athanasopoulos et al., 2007; Rae et al., 2009), causing a decrease in bone water volume and skeletal blood flow (Goans et al., 1995; Drescher et al., 2000). PEMFs exert angiogenic–osteogenic effects on bone marrow during GC treatment by maintaining this change (Wang et al., 2021; Wang et al., 2022a).

Recently, cellular senescence was demonstrated to play a fundamental role in GIOP (Liu et al., 2021; Wang et al., 2021; Wang et al., 2022c). Nestin^+^ MSCs in young mice and LepR^+^ MSCs of adult mice undergo senescence in response to GCs (Li et al., 2017; Su et al., 2020; Wang et al., 2021). Clearance of senescent cells by PEMF treatment (8 Hz, 3.8 mT, 1 h per day) for 4 weeks rescued GC-induced bone loss (Wang et al., 2021) through the EZH2–H3K27me3 axis.

Overall, GCs cause osteoporosis by inhibiting bone formation and enhancing bone resorption, which is prevented by PEMFs through different mechanisms. Thus, PEMFs should be considered a promising method for treating GIOP. Moreover, it seems that PEMFs with different parameters including frequency, intensity, and duration can still influence GIOP (Cai et al., 2020; Wang et al., 2022b). However, studies have focused on the physiological effects of PEMFs on bone cells or on other types of osteoporosis; thus, the effects of PEMFs on GIOP are still questionable. The positive effects of PEMFs on osteoporosis are still unclear due to the use of different parameters including the PEMF waveform, daily exposure time, treatment starting point and duration, and other subject-related factors. Moreover, there are great differences between clinical experiments and animal experiments. To verify these findings, more high-quality, reliable, randomized controlled trials with large sample sizes and long-term follow-up are needed in the future. In addition, the contraindications of long-term PEMFs should be considered in further studies.

## 6 Conclusion

**FIGURE 1:**
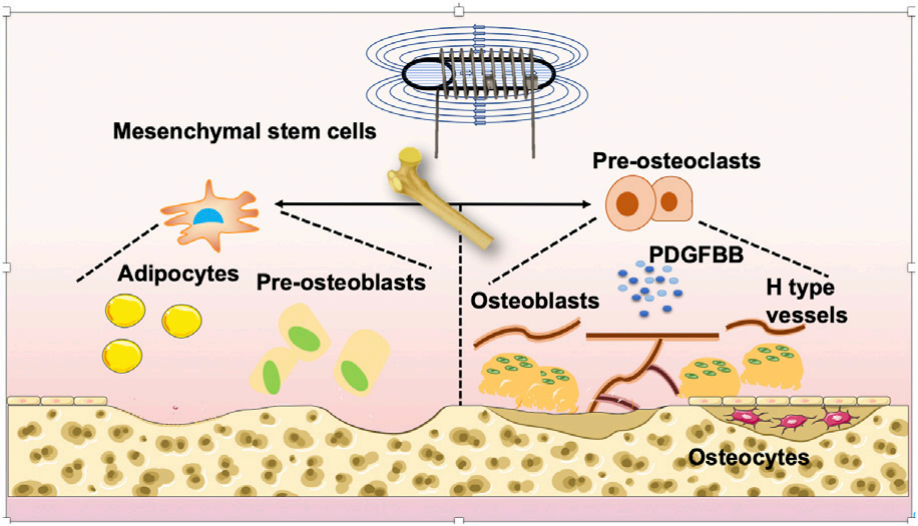
Mechanism of PEMF-treated OP. PEMFs may be considered a potential and non-side-effect therapy for GIOP. PEMFs stimulate osteoblastogenesis, suppress osteoclastogenesis, and influence the activity of bone marrow mesenchymal stem cells (BMSCs), osteocytes and angiogenesis. Finally, it leads to the retention of bone mass and strength.

**FIGURE 2:**
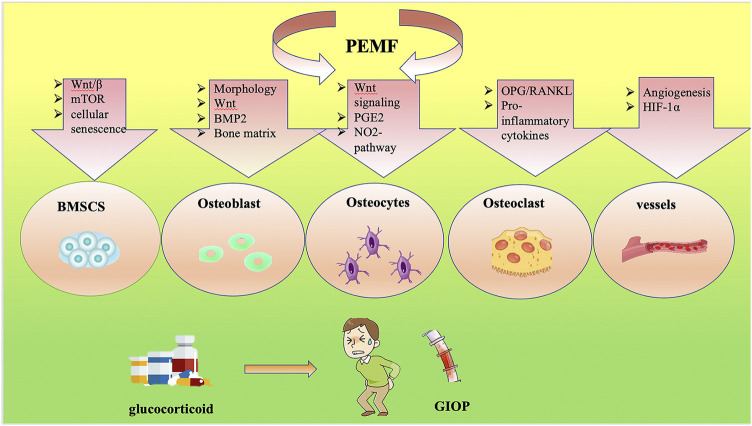
Signaling pathway of PEMF-treated GIOP.

Current studies of PEMFs and their potential roles in regulating bone metabolism in GIOP are summarized in this review (Figures 1, 2). PEMFs should be recommended based on more reliable evidence from high-quality, randomized controlled trials, which require clinical studies with large sample sizes and long-term follow-up. Moreover, gene-knockout mice should be used to determine the specific target for treating GIOP by PEMFs. After that, the usage of PEMFs can be considered a safe treatment for GIOP.
